# Giant enchondroma recurrence of the proximal phalanx of the fifth finger: a case report

**DOI:** 10.11604/pamj.2020.36.7.19186

**Published:** 2020-05-06

**Authors:** Dario Pilla, Alessandro Geraci, Lawrence Camarda, Alberto Ricciardi

**Affiliations:** 1 Orthopaedic Department, San Giacomo Apostolo Hospital, Castelfranco Veneto, Italy; 2 Department of Orthopaedic Surgery (DICHIRONS), University of Palermo, Palermo, Italy

**Keywords:** Enchondroma, hand, Tsuge technique

## Abstract

Enchondroma (EC) is a benign and cartilage-forming tumor that causes intramedullary lesions. Moreover, EC is the most common bone tumor in the phalanges and metacarpal bones of the hand, deforming the structure and causing pain and functional limitation. The management of this neoplasia is the surgical treatment and the approach that is well-accepted consists in the curettage followed by the void augmentation with biological or synthetic fillers. The results from surgery are usually good and the recurrence rate is low (2-15%). In this article we report a case of EC recurrence of the proximal phalanx of the fifth finger of the hand after curettage and grafting. The patient was treated with the amputation of the fifth ray according to the Tsuge technique, obtaining a satisfying clinical result.

## Introduction

Enchondroma (EC) is benign tumor from cartilage origin, that mainly affects the skeleton of the hand and malignant transformation of solitary enchondroma is extremely rare (< 1%) [1]. In the context of enchondromatosis (Ollier disease and Maffucci syndrome) the risk of malignant transformation is increased up to 35% [2]. Often patients with EC are asymptomatic or with few symptoms and signs such as a localized painless swelling. For this reason, EC may be diagnosed during a routine physical examination, as an incidental finding on plain radiographs or in the event of a pathologic fracture commonly caused by minor trauma and favored by the presence of the tumor [3]. Conservative treatment through regular check-up and surgical excision using curettage are the two major treatment methods for EC. Surgical treatment of this lesion is recommended for a histological diagnosis execution, as well as for the prevention of complications, such as progressive finger deformity, pathological fracture and tendon injuries [4]. Curettage and bone grafting have been the conventional methods of treatment. Other treatment approaches include curettage and filling of bone substitute, cementation and additional chemical treatment. Surgery outcome are usually successful and the recurrence rate is low (2-15%) [5]. We report a case of a giant EC recurrence of the proximal phalanx of the fifth finger of the hand (PP5th) after curettage and back-filling with calcium phosphate bone cement (CPC), treated with the amputation of the fifth ray according to the Tsuge technique.

## Patient and observation

**Figure 1:**
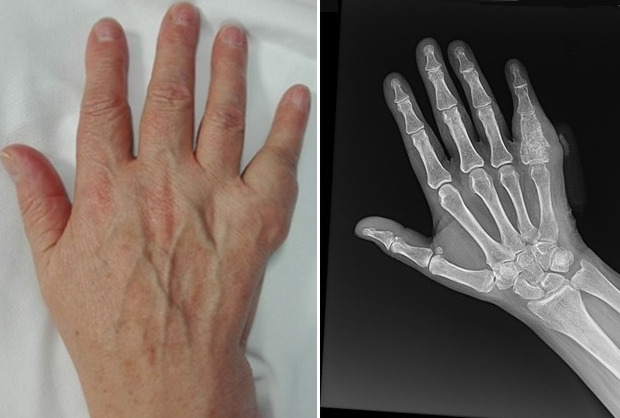
Five years after surgery, the patient returns for pain, swelling and functional limitation; this image highlights the hand and the X-ray with evident recurrence of the pathology

A 66 years-old female with no significant medical problems was examined in Orthopaedic Department of the San Giacomo Apostolo Hospital (Castelfranco Veneto, Treviso, Italy). The patient presented pain and inability to flex the 5^th^ finger of her right dominant hand. Five years earlier, the patient presented an EC in the same phalanx treated with curettage and back-filling with CPC. Two years after surgery, a radiographic evaluation was performed, disclosing good progress. The patient denied direct trauma and smoking or alcohol consumption. No risk factor was present, and no rheumatic disease or chronic joints diseases have been reported. She reports chronic pain on the 5^th^ finger of the hand with progressive enlargement of the finger, paresthesia, tactile pain and swelling of the ulnar region of the hand. On the clinical examination, a painful palpable mass on the phalanx with crepitation was observed, with a limited passive/active proximal interphalangeal joint (PIP) flexion. The volume of the PP5th was considerable (**Figure 1**). The range of movement of the PIP was 0 degree of extension and 15 degree of flexion. Tinel's Sign and Phalen's Maneuver are negative. Normal range-of-motion of the wrist was observed.

**Instrumental tests:** on anteroposterior and lateral X-ray of the fingers, a complete absorption of the CPC, with an increased dimensions of the phalanx it was noted. Radiographs revealed a well-demarcated and pathologic fracture with cortical thinning of the PP5th, with a radiolucent mass in the diaphysis of the same phalanx (Figure 1). The soft tissue mass was relatively well marginated. The hematochemical analysis excluded the presence of rheumatic disease or an increase of the uric acid levels.

**Intraoperative findings and surgical technique:** surgery was performed in accordance with the technique described by Tsuge in 1989, which consisted in the disarticulation of the fifth metacarpal, together with amputation of the fifth ray of the hand [6]. An axillary block anesthesia was carried out and a tourniquet was applied. Tumor excision was performed through a volar Z-Brunner type incision and dorsal longitudinal extension along the digit. A Y-shaped incision began dorsally at the base of the fifth metacarpal, continued in a diamond form in the dorsal space between the fourth and the fifth metacarpal and ended in a V-shape on the palmar side at the level of the proximal transverse palmar plica. The dorsal skin flap was unglued to expose the fourth dorsal interosseous and the portion of the metacarpus to be resected. After complete bone exposure, the oblique mid-diaphysis osteotomy of the fifth metacarpal was performed, leaving its base in place to maintain distal insertion of the carpal ulnar extensor.

Subsequently, the extensor muscle tendon section of the little finger and of the joint extensor tendon of the fifth finger were performed, without injuring the common extensor tendon of the fourth finger. The hypotensive intrinsic muscles were leaved, while the fifth abductor tendon was distal sectioned and sutured to the fourth dorsal interosseous muscle. After ligature of the common digital vessels at their base, the corresponding nerve endings were cauterized at the same level and pushed into the interosseous muscular tissue. Then the deep intermetacarpal ligament was incised, stretched transversely between the head of the fourth and fifth metacarpal bone. Finally, the distal portion of the fifth metacarpal bone was removed. At this point, the abductor tendon of the fifth finger was sutured on the fourth dorsal interosseous muscle, to fill the gap created by the fifth metacarpal bone removal.

**Figure 2:**
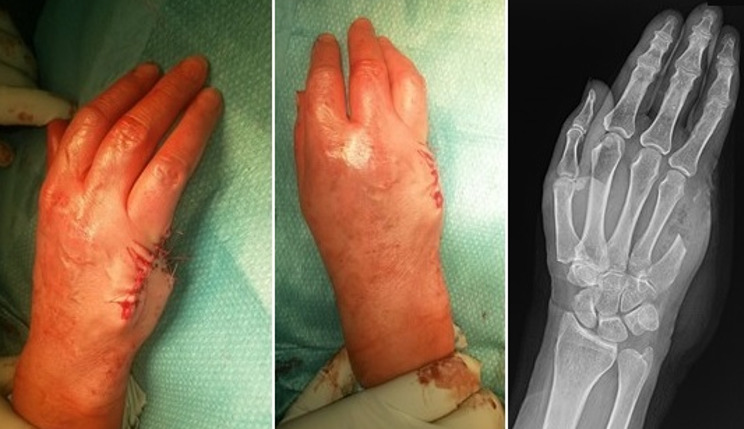
Surgical and radiographic result immediately after surgery

**Figure 3:**
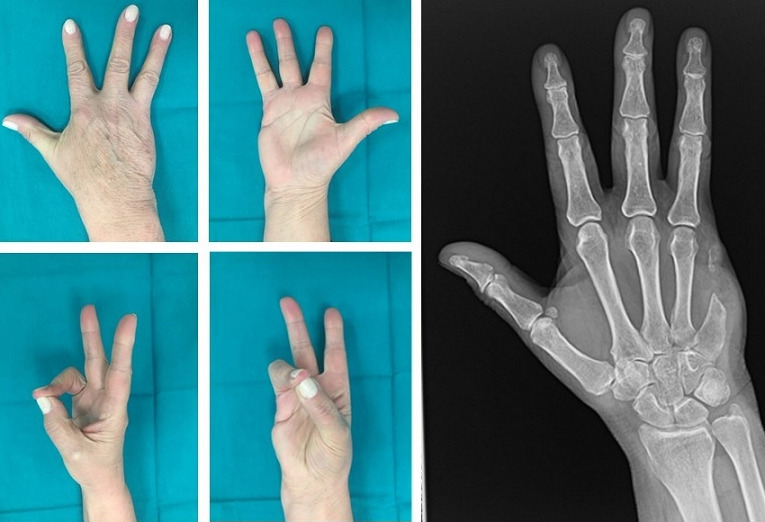
Clinical and radiographic result after one year from surgery

The skin flap was sutured first to the palm. Secondary, the skin was sutured to the dorsal hand where the skin, thin and mobile, is more easily modeled (Figure 2). A soft bandage was then applied to the patient´s forearm with the fourth and third fingers included. Functional rehabilitation program started at two weeks post-operatively and it was designed in order to increase the range of motion and the strength of the hand grip. Histological analysis revealed a benign expansive EC of the hand. The patient resumes her job at the factory after 2 months. One year after surgery, clinical and a radiographic assessment were carried out. The patient reported the absence of any subjective problems, with complete functional recovery of the operated hand. She was satisfied with the cosmetic results that had been achieved and the hand functionality (Figure 3).

## Discussion

EC is a relatively benign medullary cartilaginous neoplasm with benign imaging features. In diaphyseal localizations the treatment of choice remains the complete emptying of the lesion, through a “window of the cortical bone” and subsequently curettage and grafting with allogenic bone, autogenous bone or synthetic bone substitutes [7]. Various recurrence rates were reported in literature with different treatments. In the retrospective review on 102 patients, Sassoon *et al.* observed a recurrence rate of 6% [8]. Whereas, Gaulke and Suppelna reported a recurrence rate of 14% in a long-term follow-up (mean, nine years) and all recurrences (3 patients) were discovered after 10 years from the surgery [9]. Further, Wolf *et al.* detected the presence of recurrence of 6.5% after treatment with curettage and application of CPC [10]. Amputation is performed when the EC evolves into chondrosarcoma, or in case of multiple relapses, in order to prevent malignant evolution of the disease.

In addition, amputation can be considered when bone deformity becomes so evident to compromise joint´s function. In our case, the patient has an EC recurrence after 5 years from the surgical treatment. Because of pain and functional limitation of the fifth finger, we decide to perform an amputation of the fifth ray. Indeed, the lesion´s size prevented the correct movement of the fifth finger and the function of the whole hand. Amputation seemed to us to be the best choice to eradicate pain and prevent a possible new recurrence or malignant evolution. With regards to the best level at which surgical amputation should be performed, on both functional and cosmetic grounds, the complete removal of the fifth ray is preferable to the reshaping of the stump or to the disarticulation at the level of the metacarpophalangeal joint. An amputation abutment of the little finger at the level of the first phalanx has no functional significance and is unsightly. Moreover, the stump tends to be a hindrance in normal daily activities (i.e.: open and close a drawer, handle domestic utensils).

## Conclusion

The simple curettage represents an effective option for the treatment of most hand EC. When recurrence affects the PP5th, the simple amputation of the finger can create difficulty on the functionality of the hand. As an alternative the amputation might include parts of the fifth metacarpal bone in order to improve hand movement and pain.

## Competing interests

The authors declare no competing interests.
